# Testing and Optimizing a Stove-Powered Thermoelectric Generator with Fan Cooling

**DOI:** 10.3390/ma11060966

**Published:** 2018-06-07

**Authors:** Youqu Zheng, Jiangen Hu, Guoneng Li, Lingyun Zhu, Wenwen Guo

**Affiliations:** 1 Department of Energy and Environment System Engineering, Zhejiang University of Science and Technology, Hangzhou 310023, China; zyq888@zust.edu.cn (Y.Z.); 211601802004@zust.edu.cn (L.Z.); guowenwen@zust.edu.cn (W.G.); 2 Hangzhou YiNeng Power Technology Corporation Limited, Hangzhou 310014, China; hjg2623@sina.com

**Keywords:** thermoelectric generator, power load feature, thermoelectric efficiency, optimization

## Abstract

In order to provide heat and electricity under emergency conditions in off-grid areas, a stove-powered thermoelectric generator (STEG) was designed and optimized. No battery was incorporated, ensuring it would work anytime, anywhere, as long as combustible materials were provided. The startup performance, power load feature and thermoelectric (TE) efficiency were investigated in detail. Furthermore, the heat-conducting plate thickness, cooling fan selection, heat sink dimension and TE module configuration were optimized. The heat flow method was employed to determine the TE efficiency, which was compared to the predicted data. Results showed that the STEG can supply clean-and-warm air (625 W) and electricity (8.25 W at 5 V) continuously at a temperature difference of 148 °C, and the corresponding TE efficiency was measured to be 2.31%. Optimization showed that the choice of heat-conducting plate thickness, heat sink dimensions and cooling fan were inter-dependent, and the TE module configuration affected both the startup process and the power output.

## 1. Introduction

**Figure 1:**
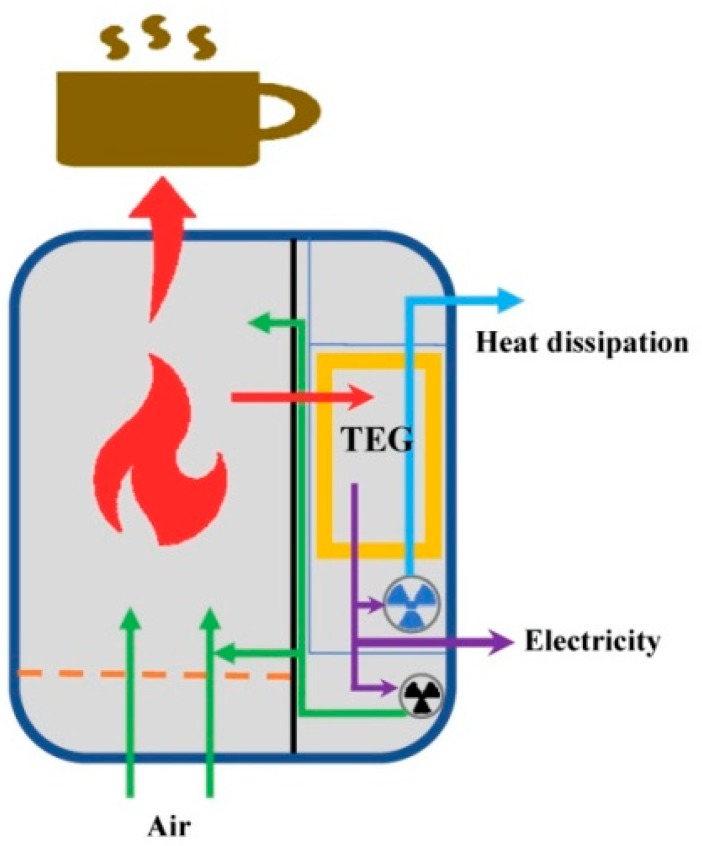
Diagram of a basic stove-powered thermoelectric generator (STEG).

One-point-three billion people still live in off-grid areas [1], and natural disasters often cut off the electricity supply in developed countries and regions. Providing a minimum amount of electricity in an off-grid area and under emergency conditions is vital for communications, medical electronic devices, lighting and other basic needs. A primary battery is the best choice, yet it has a limited life and the risk of unavailable resupply. Solar power generators and wind power generators are major solutions for the off-grid regions, yet they are weather dependent. Hand generators could be the ultimate solution, but depend on manpower. Therefore, other technologies should be developed to provide a minimum amount of electricity. Generating electricity from biomass stoves has attracted much attention in recent years [2]. This has been achieved by adopting TE modules so that combustion inside the stove can power the thermoelectric generator, while cooking and heating still work simultaneously. Over two billion people burn biomass for cooking and heating [3], which means that the stove-powered thermoelectric generator (STEG) has good prospects. Further, a well-designed STEG can power a blower to improve the combustion inside the stove, reducing CO and particle pollution [4], which are known to be harmful. Figure 1 shows a basic STEG in diagram form.

The STEG utilizes the Seebeck effect, i.e., the temperature difference forces electrons to move in one direction between two different metals or semiconductors [5]. The major advantages of a STEG are little maintenance and weather independence, whereas its drawback is low efficiency [6,7], a problem that may be solved by new technologies based on future research [7]. Many previous studies have focused on STEG, but only a few selected experimental studies are reviewed in the present work. Experimental work on STEG falls into three groups: water-cooled STEG, natural draft air-cooled (NDAC) STEG and forced draft air-cooled (FDAC) STEG.

For the water-cooled STEG, Rinalde et al. [8] obtained a total of 10 W of electricity. No DC-DC converter was used, and the power consumed by the water pump was not considered. Champier et al. [9,10] designed a STEG to produce electricity and to improve the combustion efficiency. The cold end was cooled by a water tank, and an output of electricity of 6 W was recorded [9]. In this work, a DC-DC converter was used, and the TE efficiency was found to be about 2% for a temperature difference of 200 °C. For the optimized STEG, the same cooling method was used, but the output power increased to 7.6 W [10]. Montecucco et al. [11,12] designed another type of water-cooled STEG, producing a net output of electricity of 19 W with a TE efficiency of about 4–5% for a temperature difference of 150 °C–200 °C. A DC-DC converter was employed here.

For NDAC STEG, Nuwayhid et al. demonstrated that the combination of a TE module and a stove can produce electricity [2,13,14]. The STEG was optimized several times to increase the output power from 1 W [2] to 3.4 W [13], and then to 4.2 W [14]. The cooling method was natural air convection, using ordinary finned heat sinks or heat pipes, and no DC-DC converter was employed. Lertsatitthanakorn [15] developed a STEG with a maximum of 2.4 W recorded. The power load feature and the TE efficiency for various temperature differences were studied, indicating that the load resistance should be optimized to maximize output power, while the TE efficiency ranged from 1%–3.2% when the temperature difference varied from 44 °C–150 °C. Najjar and Kseibi tested a novel STEG to produce hot water and electricity [16,17]. A maximum power output of 7.8 W was recorded with no DC-DC converter. Detail temperature distributions were measured in different positions, and the influence of different fuel types was explored in these works. Moreover, a detailed comparison of previous STEGs was presented in their work [17].

For FDAC STEG, Mal et al. [4] tested a STEG, improving the exhaust gas quality (CO and particles) by adding a blowing fan. It was found that the CO and particle concentration decreased significantly when using a blowing fan; while the STEG produced electricity between 2 W and 4 W. O’Shaughnessy et al. [18] distributed several STEGs in off-grid regions and ran a field test for 80 days. It was found that 3 Wh of electricity power met the basic need. Recently, the BioLite CampStove and BaseCamp, designed for outdoor activities and emergency conditions, can provide 2–5 W of electricity [19]. Batteries are incorporated and should be charged before first use.

All the above works conclude that the STEG offers a suitable and economical alternative way to produce electricity in off-grid areas and under emergency conditions. However, different opinions should not be ignored, e.g., Sornek et al. [20] concluded that it is not an economical method of producing electricity using TE modules, and the payback period is too long.

Surveying the above literature, while many aspects of various types of STEG were studied, indicating that the STEG is a potential method of obtaining electricity in off-grid areas and under emergency conditions, as of yet, it is far from fully understood. Several conclusions can be drawn based on the above literature.

(1) Water-cooled STEGs have larger output power than air-cooled ones. However, water is not always available everywhere at all times.
(2) NDAC STEGs were mostly used in previous studies, resulting in relatively large volumes and weights.
(3) FDAC STEGs have attracted attention recently, yet studies are limited.
(4) For FDAC STEGs, only a limited number of TE modules can be installed, which restricts their application.

In the present work, an FDAC STEG was designed with a novel type of heat collector allowing the installation of as many as eight TE modules. For safety and ease of use, no battery was incorporated. The maximum electricity power output is 60% larger than that of the available commercial product under comparable weight [19]. First, the structure of the STEG and the experimental system are discussed. Second, the results of the startup performance, power load feature and TE efficiency are presented and discussed in detail. Third, the heat-conducting plate thickness, cooling fan selection, heat sink dimensions and TE module configuration are optimized. Finally, several conclusions are drawn. The present study offers new experimental data on FDAC STEGs and presents a new type of STEG.

## 2. Experimental System

**Figure 2:**
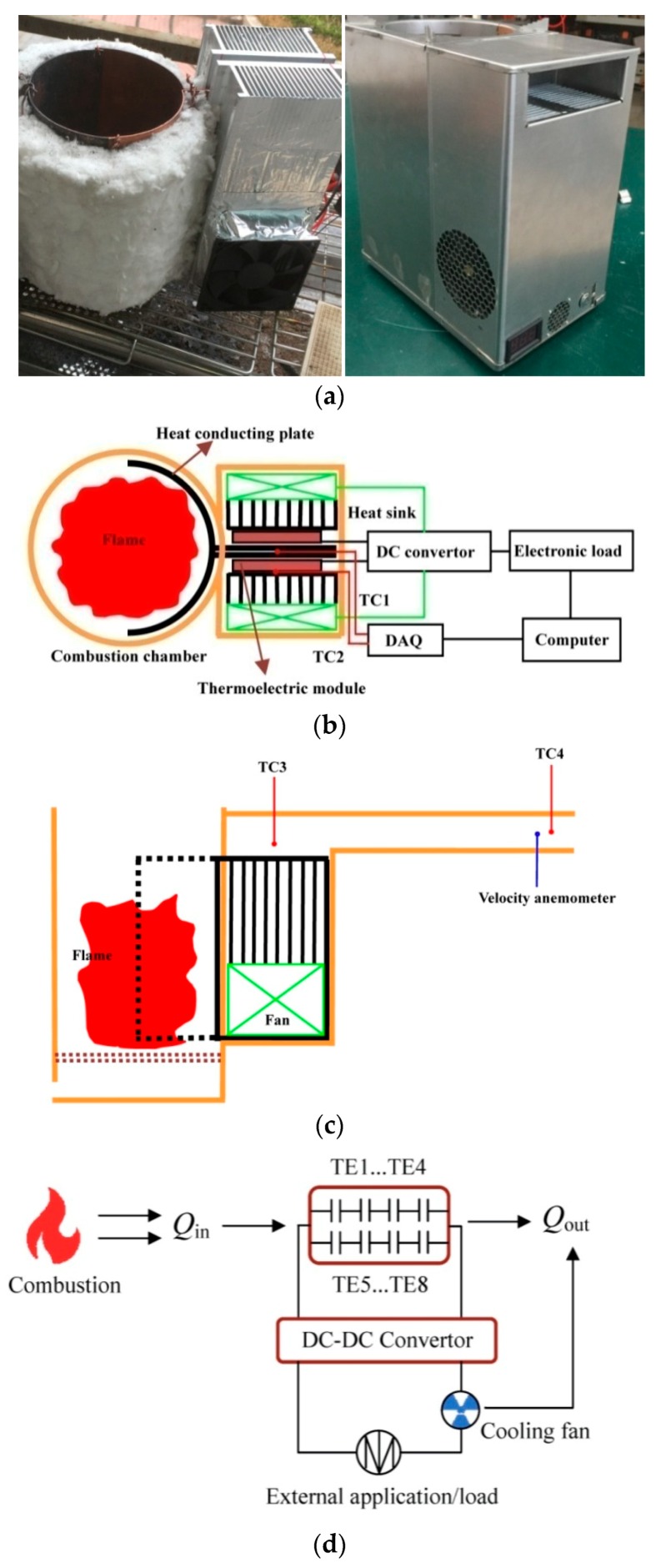
The STEG photographs, the experimental setup and the electric circuit (**a**) photographs; left: STEG core, right: a pilot product; (**b**) a sketch of the experimental setup (top view); (**c**) a sketch of the experimental setup (side view); (**d**) schematic drawing of the electric circuit with multiple TE modules fitted.

The experimental setup and the electricity circuit are shown in Figure 2. The STEG consists of a semi-circular steel plate with a porous fuel holder, two quarter circle copper heat-conducting plates, eight TE modules, two aluminum alloy heat sinks, two cooling fans and two DC-DC converters. The semi-circular steel plate and the two quarter circle heat-conducting plates are installed together to form a circular combustion chamber, while a 30 mm-thick layer of fiber glass insulation is wrapped round the outside of the combustion chamber. The diameter and the height of the combustion chamber are 140 mm and 220 mm, respectively, and a porous fuel holder is installed 30 mm above the bottom surface of the combustion chamber. The copper heat-conducting plate has a “Z” shape, and two plates are installed together in opposing direction to form a semi-circle to provide part of the combustion chamber and to form a flat plate for the installation of the TE modules. Eight TE modules, type “TEP1-126T200” with dimensions of 40 mm (length) × 40 mm (width) × 3.8 mm (thickness), are installed on the opposing surfaces of the right-hand side of the heat-conducting plate, equally spaced. The TE material is Bi_2_Te_3_, and the dimensions of the thermo-element (leg) are 1.3 mm × 1.3 mm × 1.55 mm (length). Typically, the TE module produces 2.8 watts under a temperature difference of 150 °C, and the working temperature of the TE module does not exceed 250 °C. Two aluminum alloy heat sinks are bolted above the TE modules. Two DC air fans are installed on the top surface of the heat sinks, blowing outside air into them, while aluminum foil was used to seal the top surface of the other heat sinks. In order to determine the TE efficiency, an extensible aluminum tube with a diameter of 120 mm was installed to connect the outlet of the heat sinks, so as to form an air duct. This extensible aluminum tube can be used to supply clean and warm air to tents, and heat insulations should be employed when the distance between the tent and the STEG is far away. Two DC-DC converters (type MP1583) were adopted to stabilize the output voltage at 5.0 V. One DC-DC converter was used to supply electricity to the cooling fans, and the other one was used to provide electricity for the external load.

A sketch map of the experimental system is shown in Figure 2b,c. Four thermocouples with a diameter of 1 mm are installed. The first thermocouple is installed at the center point of the top end of the heat-conducting plate, measuring the hot end temperature. The second one is installed at the center point of the top end of the heat sink, measuring the cold end temperature. The third thermocouple is installed at the outlet of the heat sink, while the fourth one is installed at the exhaust exit of the extensible aluminum tube. The distance from the outlet of the heat sink to the exhaust exit is 800 mm, and screens are installed to ensure uniform air flow. The measuring range and the accuracy of the thermocouples are −200–400 °C and ±0.5%, respectively. The temperature signals were recorded by an Agilent-34,970 A data acquisition instrument combined with a Benchlink Data Logger program. The average air velocity was measured with a Peakmeter MS6252B turbo type anemometer. The measuring range and the accuracy of the thermo-anemometer are 0.8–30.0 m/s and ±2.0%, respectively. The average air velocity and the exhaust air temperature (*T*_4_) are used to calculate the mass flow rate of the cooling air. The power load feature was measured using Prodigit 3311F electronic load. Its measuring range and accuracy are 0–60 V (300 W) and ±0.5%, respectively. Charcoal is used as the fuel in the present experiment. The net calorific power and the density of the charcoal is 31.2 MJ/kg and 1322 kg/m^3^, respectively, and the ash mass fraction is 4.87%. The errors of the parameters are shown in Table 1, where the efficiency of the DC-DC converter was found to be 77.3% using two electric energy testers installed before and after the DC-DC converter. The operation procedure of a running of the present STEG test includes several steps. e.g., Step 1: Put a certain amount of dry branches into the combustion chamber, then put a certain amount of charcoal above the dry branches. Turn on the data acquisition instruments, and initialize the data recording programs. Step 2: Ignite the dry branches. Step 3: Carry out the power load tests when the STEG reaches the steady state. Step 4: Hard charcoal is added into the combustion chamber if necessary. Step 5: Measure the parameters of the exhaust flue gas. On the other hand, the typical running of the pilot product based on the present STEG shown in Figure 2a is much more user-friendly. First, ignite the branches or any other combustible solid fuels. Second, the clean-and-warm air and electricity can be used when the indicator is above 15 within minutes (an indicator was designed in the pilot product).

**Figure 3:**
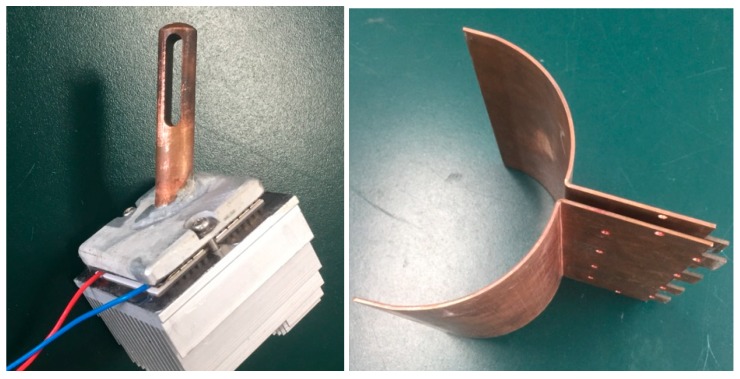
The heat collector. Left: copper rods inserted into the flame zone (a photograph of part of the CampStove [19]); right: copper plates forming part of the combustion chamber in the present unit.

Figure 3 shows the heat collector. Copper rods are installed in earlier FDAC STEGs [4,18,19], inserted into the fame zone. This design may be inconvenient when adding fuels, and gaps between the copper rods and the plate where TE modules are fitted may cause the failure of the STEG [18]. In the present unit, copper plates are used as the heat conductor. This avoids possible gaps. This type of heat collector has several advantages: (1) It is easier to install several TE modules. (2) There is no intrusion into the combustion chamber. (3) It works with various types of fuel, i.e., flaming fuels such as dried twigs, and flameless fuels, such as charcoal. The disadvantage is the weight of the heat collector. A pilot product with dimensions of 265 mm × 173 × 320 mm, based on the present STEG, is shown on the right side of Figure 2a, and it has all functions indicated in Figure 1. The mass weight of the pilot product is 8.42 kg, which is 0.26 kg heavier than the BioLite BaseCamp [19].

## 3. Results and Discussions

### 3.1. Startup Performance

**Figure 4:**
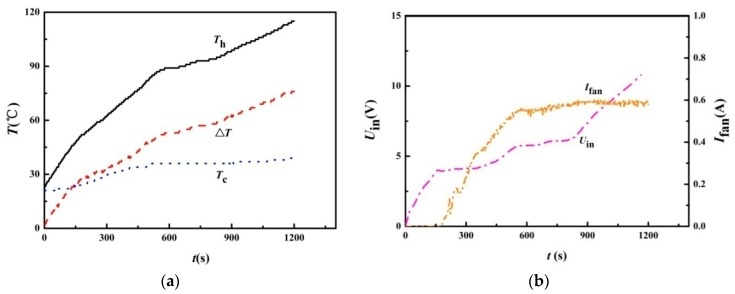
The self-startup process of the STEG. (**a**) The hot end temperature, *T*_h_, cold end temperature, *T*_c_, and temperature difference, Δ*T*; (**b**) the closed circuit input voltage, *U*_in_, and the fan current, *I*_fan_ (Cod1 heat conducting plate, HS1 heat sink, YNJAD the cooling fan and Cof1 the TE module configuration).

A typical startup process is shown in Figure 4 with the atmospheric temperature lying between 21 °C and 22 °C during the experiments. After ignition, heat will be conducted to the hot end of the TE modules, increasing the hot end temperature. Meanwhile, heat conducted through the TE module reaches the finned heat sinks. The heat capacity of the heat sinks results in a certain temperature difference, which produces voltage by the TE modules. After a certain time (218 s), the cooling fan begins to work. This is similar to the result of a previous test [4], which indicated that it takes 120–300 s for the cooling fan to self-startup. The self-startup time depends on the flame intensity inside the combustion chamber and the heat capacity of the heat sink. The hot end temperature, *T*_h_, cold end temperature, *T*_c_, temperature difference, Δ*T*, closed circuit input voltage, *U*_in_, and fan current, *I*_fan_, are shown in Figure 4. After 850 s, the cooling fans maintain at the normal operating speed, then the input voltage undergoes a rapid increase, indicating extra electricity is produced and available.

The electricity consumed by each cooling fan is 1.59 W, which is comparable to that of [4], and it is larger than that of the CampStove [19]. Note that the CampStove and BaseCamp [19] are fitted with a Li-ion battery, which provides electricity to the cooling fan during startup. These have to be charged before initial use. One advantage of the FDAC STEG is its compact volume and portable weight. However, its output power is low. The electricity consumed by the cooling fans in the present unit, *P*_fan_, is calculated by:

(1) $$P_{\mathrm{fan}}=\frac{U_{\mathrm{ou}t}I_{\mathrm{fan}}}{\xi_{\mathrm{DC}}}$$

where *U*_out_ is the output voltage (5.2 V) of the DC-DC converter, and the total *P*_fan_ equals 4.1 W. As a result, it needs about 850 s to startup, and all the electricity generated by the TE modules is consumed by the cooling fans during startup. Therefore, a battery is needed by both CampStove and BaseCamp [19] to start the cooling fan, or the heat sink should be designed to have enough heat capacity [18], which means more weight and volume. An alternative way is to include more TE modules, as in the present unit. Therefore, a novel heat collector (shown in Figure 3) is designed to include as many as eight TE modules. The disadvantage of the present STEG is higher cost, but it has larger output power, and it is not fitted with a battery, ensuring a long life. As shown in Figure 4, the corresponding temperature difference is 60 °C at 850 s, which means that a larger temperature difference is needed in order to extract electricity. 

### 3.2. Power Load Feature

**Figure 5:**
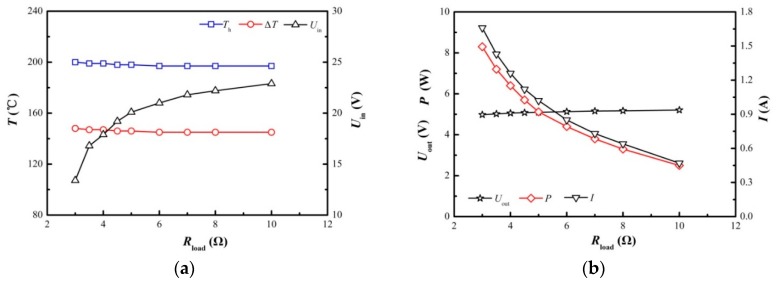
The power load feature of the STEG with a DC-DC converter. (**a**) The hot end temperature, *T*_h_, temperature difference, Δ*T*, and the closed circuit input voltage, *U*_in_; (**b**) the output voltage, *U*_out_, the load current, *I*, and the electric power output, *P*. (Cod1 heat conducting plate, HS1 heat sink, YNJAD cooling fan and Cof1 TE module configuration).

It is widely held that a power load test should be performed to find the maximum output power, and several previous studies have carried out such tests [2,9,15]. However, to the best of the authors’ knowledge, no previous studies have tested the power load feature when adopting a DC-DC converter. A DC-DC converter is important since the external load, such as batteries, lamps and electronic devices, have to work within a certain voltage range. The present work tries to probe for some understanding of this matter. The DC-DC converter used in the present unit maintains an output voltage of 5.0 V, yet the output voltage will fall if the input voltage is too low, i.e., the input voltage has to be 0.5 V higher than the output voltage. Therefore, a workable output voltage range is defined as 4.9 V < *U*_out_ < 5.0 V, and the electric power output should last at least 15 min after adding the load. Results are shown in Figure 5, presenting the hot end temperature, *T*_h_, cold end temperature, *T*_c_, temperature difference, Δ*T*, output voltage, *U*_out_, load current, *I*, and the electricity power, *P*, under different load resistances, *R*_load_.

Surveying previous studies, it is obvious to conclude that there is a critical load resistance to extract maximum electric power [2,9,15], i.e., when the load resistance equals the internal resistance of the TE modules. For example, the maximum *P* equals about 0.8 W when *R*_load_ = 3 Ω (Δ*T* = 68 °C) in [2], while the maximum *P* equals 1.0 W when *R*_load_ = 1.7 Ω (Δ*T* = 99 °C) in [9], and the maximum *P* = 2.4 W when *R*_load_ = 7 Ω (Δ*T* = 150 °C) in [15]. For load resistances smaller than the critical one, the output electricity remains, even though it is smaller than the maximum electric power [2,9,15].

When using a DC-DC converter, as shown in Figure 5b, lower load resistance results in larger electric power outputs, or the DC-DC converter fails to maintain a constant voltage when the load resistance is too low, which means the whole STEG will crash. The crash is caused by the low output voltage since it is directly affecting the working speed of the cooling fans. As shown in Figure 5, all the experiments were conducted at a comparable temperature difference, i.e., 145 °C ≤ Δ*T* ≤ 148 °C, and atmospheric temperature between 21 °C and 22 °C. The STEG requires a minimum load of 3 Ω to function fully. The corresponding electric power output is 8.25 W, which is greater than that of BaseCamp [19]. The shell of a pilot product based on the present STEG unit has a minor effect on the electric power output. Tests showed that the pilot product of the STEG is still able to supply over 8.0 W of electric power. Another phenomenon is that the electric power output decreases rapidly with the load resistance, e.g., *P* = 8.25 W when *R*_load_ = 3 Ω and *P* = 2.5 W when *R*_load_ = 10 Ω. Therefore, a suitable load is critical to extract maximum electric power. With regard to a single TE module’s output, the present STEG is able to generate electric power:

(2) $$P_{\mathrm{TE}}=\frac{P_{\max}}{N\xi_{\mathrm{DC}}}+\frac{P_{fan}}{N}=1.85\;W$$

where *N* is the number of TE module installed in the STEG. This is comparable to the CampStove and BaseCamp [19] and is 0.55 W less than that of [15] (water-cooled STEG) at the same temperature difference.

The maximum power point tracking (MPPT) DC-DC converter is widely used in solar power generation [21,22]. This technology was employed in TE generators [23,24,25] and was adopted in Champier’s work [10] and Montecucco’s works [11,12] in STEG studies. In the present work, a regular DC-DC converter was chosen instead of an MPPT DC-DC converter. The present STEG offers electric power output as high as 1.6 A at 5.0 V, which is enough for most available USB devices. USB devices have their own power manager, and their charging current is controlled (mostly limited to 1.0 A). For some USB devices charging with 2 A, their power managers were designed to be self-adapting between 1 A and 2 A. There is no doubt that the MPPT DC-DC converter helps to extract the electricity as much as possible. However, it has to work with a battery, and the battery should accept all the provided electric energy. In case the electric power output is large enough for USB devices, the MPPT DC-DC converter is no longer needed. This may lead to a certain waste of electric power, yet the STEG becomes easier to use. Users do not have to charge the battery for maintenance and before use. Meanwhile, users do not have to wait before the STEG is ready for electricity output. For future STEGs that have to incorporate a battery, an MPPT DC-DC converter should be used. The present STEG is ready to adopt a mating combination of an MPPT DC-DC converter and a battery. A tunable MPPT DC-DC converter can be obtained widely on the open market.

### 3.3. TE Efficiency

In order to determine the TE efficiency, the amount of heat dissipation by the heat sinks is calculated approximately, according to the following equations [26],

(3) $$Q_{\mathrm{out}}=c_{p}m_{\mathrm{ex}}(T_{\mathrm{out},\mathrm{ave}}-T_{\infty })$$

(4) $$m_{\mathrm{ex}}=0.25\pi d^{2}V_{\mathrm{ex},\mathrm{ave}}\rho_{\mathrm{ex},\mathrm{ave}}(T_{\mathrm{ex},\mathrm{ave}})$$

Therefore, the TE efficiency can be derived from the following equations [26],

(5) $$\xi =\frac{P_{\mathrm{tot}}}{P_{\mathrm{tot}}+Q_{\mathrm{out}}}$$

(6) $$P_{\mathrm{tot}}=NP_{\mathrm{TE}}$$

Therefore, the total electric power is about 14.79 W. The average exhaust air velocity from the heat sink is 1.40 m/s, and the average exhaust air temperature is approximately 50.5 °C, while the exhaust pipe diameter is 120 mm. Therefore, the air mass flow rate from the heat sinks can be derived (17.3 g/s). The average outlet air temperature of the heat sinks was 58.0 °C, and the atmospheric air temperature was 22.0 °C during the experiments. As a result, the amount of heat dissipation by the heat sinks is approximate 625.9 W. Finally, the TE efficiency is calculated to be 2.31% at a temperature difference of 148 °C. The measured data of the flue gas are summarized in Table 2.

One advantage of the present STEG is the possible combined heat and power (CHP) application, which is proposed by water-cooled STEG studies [8,9,10,11,12]. Few previous FDAC STEG studies proposed a CHP design, while all the previous FDAC STEGs used the heated air as the combustion air. As shown in Table 2, the TE efficiency is around 3% for available STEGs. Therefore, enough TE modules have to be employed in order to design a CHP application, while the heat collector is the essential issue in order to install several TE modules. The present work proposed a novel heat collector to install as many as eight TE modules. Therefore, the heat dissipation power is 625 W, which is transferred to the clean air. As a result, a CHP application is obvious. Tests showed that the present STEG can warm a double resident tent from 5 °C–22 °C after 20 min of heating when the ambient temperature is 5 °C. The present STEG is able to provide warm air as long as 3.0 h when burning an amount of 0.85 kg of charcoal. As shown in Figure 1, the combustion air was supplied by another blower. The heating problem for tents is difficult to solve by solar power generation and a high-power electrical heater, since a large amount of electricity is not available for many tents.

The TE efficiency can be approximately derived theoretically by [26]:

(7) $$\xi_{\mathrm{theo}}=\frac{T_{h}-T_{c}}{T_{h}}{\left\{{\left(1+2rw\right)}^{2}\left[2-0.5\left(\frac{T_{h}-T_{c}}{T_{h}}\right)+\left(\frac{4}{ZT_{{}_{h}}}\right)\left(\frac{1+n/L}{1+2rw}\right)\right]\right\}}^{-1}$$

where *r* is the ratio of thermal contact, *w* is the ratio of ceramic thickness to that of the thermo-element and *n* the ratio of electrical resistivity. For Bi_2_Te_3_-based TE modules and the TE module dimensions of the present unit, *w* = 0.516. Both *r* and *n* are estimated to be 0.1, where *n* is in millimeters. *L* is the length of the thermo-element (leg), and *Z* is the TE figure-of-merit, which is estimated to be 1.0 × 10^−3^ K^−1^ [26]. For the parameters shown in Table 2, *T*_h_ = 473 K, *T*_c_ = 325 K. As a result, the TE efficiency is estimated to be 2.57%. The *Z* value of Bi_2_Te_3_ is well recognized, yet it is possible that *r* and *n* may vary from one manufacturer to another. Statistical studies show that the theoretical TE efficiency varies between 2.20% and 2.79% when 0.05 ≤ *r* ≤ 0.2 and 0.05 ≤ *n* ≤ 0.2, which is believed to be the case for most available Bi_2_Te_3_-based TE modules [26]. Therefore, the experimental TE efficiency agrees well with the theoretically predicted value.

The TE efficiency of various STEG is shown in Table 3, which shows that the low efficiency of STEG is the major problem for large-scale applications. 

### 3.4. Influence of the Heat-Conducting Plate’s Thickness on the Output Power

The advantage of the present heat collector is that it provides enough space to install as many as eight TE modules, and its temperature controlling method is to incorporate a copper plate with an appropriate thickness. This temperature controlling method, i.e., controlled heat flux, was widely used in earlier STEGs [4,18,19]. Copper plates with different thicknesses, shown in Table 4, were tried to explore the influence of the thickness on the output power and the temperature level. Results indicated that the 1.8-mm copper plate caused excessive temperature for dried twigs, i.e., temperatures may exceed 250 °C, which is not allowed for the present TE modules. For the present STEG, it has to be able to work steadily using various solid fuels, such as dried twigs, dried leaves, charcoals or cattle manure (nomadic people). For the above solid fuels, tests found that the burning of dried twigs in the present STEG resulted in the highest temperature level. Burning charcoal provided a smooth, steady temperature level, so it was used as the fuel in the present work.

**Figure 6:**
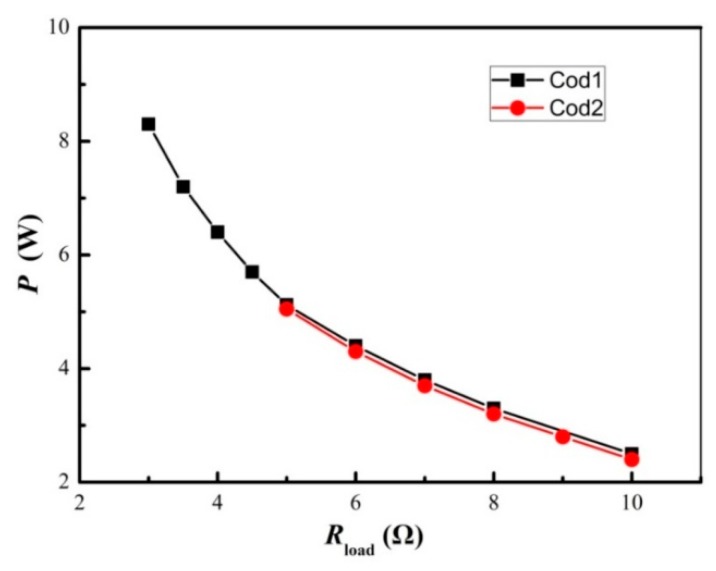
The power load feature of the STEG for different thicknesses of the heat conducting plate (HS1 heat sink, YNJAD cooling fans and Cof1 configuration).

Results of adopting the 1.5-mm copper plate and 1.2-mm copper plate are shown in Figure 6. As shown, the heat flux of the 1.2-mm copper plate is not enough for the present STEG, resulting in relatively low hot end temperatures (about 180 °C), which reduces the electric power output. For the copper plate of a thickness of 1.5 mm, tests showed that it can work steadily for various solid fuels. The maximum electric power output with the 1.5-mm copper plate is 64% higher (3.25 W) than that with the 1.2-mm copper plate. Therefore, a copper plate with a thickness of 1.5 mm is appropriate for the present combination of TE modules and heat sinks. For the present type of STEG, statistical analysis shows that the heat flux, based on the copper plate’s cross-section, should be less than 3.6 × 10^5^ W/m^2^ per TE module in order to avoid excessive temperature levels. Further, the heat flux should be greater than 2.4 × 10^5^ W/m^2^ per TE module in order to augment the electric power output. A heat flux of 2.9 × 10^5^ W/m^2^ per TE module is suggested.

### 3.5. Influence of Heat Sink Dimension on the Output Power

**Figure 7:**
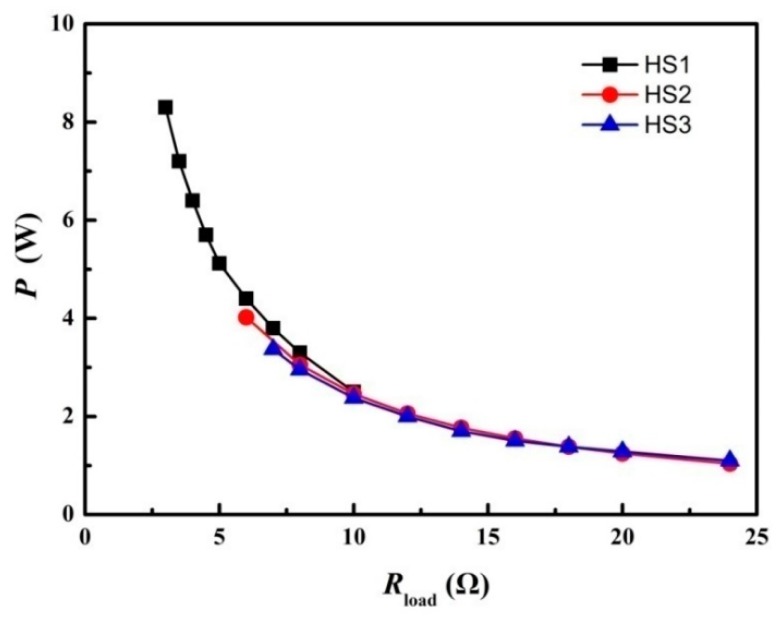
The power load feature of the STEG as different heat sinks were used.

Finned heat sinks are ordinary heat dissipating devices and are the key component in creating a temperature difference in a STEG. Several finned heat sinks with a similar total fin cross-section area (59.2 cm^2^–68.6 cm^2^), shown in Table 5, were tried to investigate their influence on the electric power output. The width of the TE module is 40 mm, and the diameter of the bolts should be over 4 mm, while enough space should be left to drill holes in the heat sinks in order to assemble the STEG. Therefore, the minimum width of the heat sink is 60 mm. The optimizing results are shown in Figure 7. As shown, for HS2 and HS3 with similar fin height and similar fin area, the maximum electric power output with HS2 is 19.3% (0.65 W) higher than that with HS3, indicating that the fin gap may be important since it is related to the wind resistance. For HS1, which has increased fin area and fin gap, the electric power output is augmented significantly. The electric power output is double that of HS2. When optimizing the heat sink, weight has to be considered, not just heat dissipating capability. For the pilot product whose STEG core is the present unit, shown in Figure 2a, the electric power output is 60% larger than that of BaseCamp [19] for a comparable weight (3.2% heavier than BaseCamp); therefore, no further optimization of the heat sink was carried out.

Limited conclusions can be drawn from the optimization of heat sinks. The heat dissipation rate of the heat sink should be less than 745 W/m^2^, while the fin gap is important for decreasing the wind resistance.

### 3.6. Influence of Cooling Fan Selection on Startup Performance and Power Output

For FDAC STEGs, the selection of the cooling fan may affect the startup process and the electric power output. Three different types of cooling fans, with dimensions of 9 cm × 9 cm × 2.5 cm, shown in Table 6, were tested to optimize the cooling effect. It should be noted that the air flow rates and the power consumed in Table 6 were measured with a Peakmeter MS6252B turbo type anemometer and an ammeter after fitting the cooling fans with HS1 heat sinks and under operating conditions. All three cooling fans have double ball bearings, yet minor differences may still exist.

**Figure 8:**
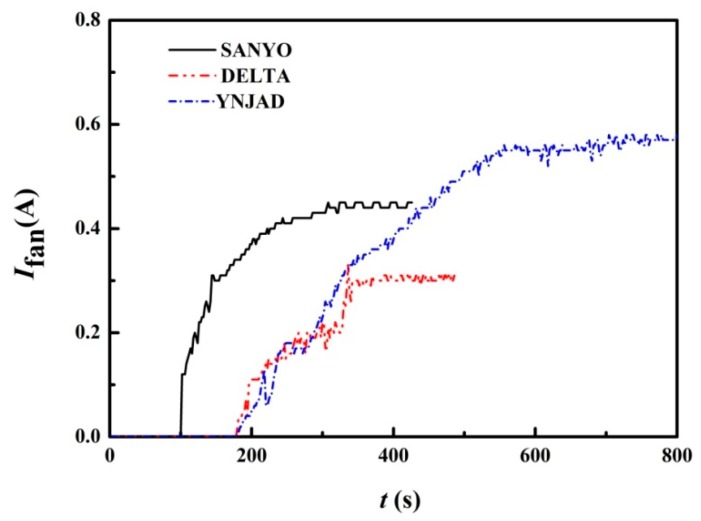
Influence of cooling fan selection on the STEG startup performance (HS1 heat sink, Cod1 heat conducting plate and Cof1 configuration).

The DELTA and YNJAD cooling fans have similar startup performance, i.e., about 200 s are needed for self-startup, as shown in Figure 8. However, the self-startup of SANYO cooling fans is much faster, needing only about 110 s. On the other hand, DELTA and SANYO cooling fans need about 350 s to reach normal operating speed, but YNJAD cooling fans need about 700 s. Therefore, the SANYO cooling fan is a promising candidate when startup speed takes priority.

**Figure 9:**
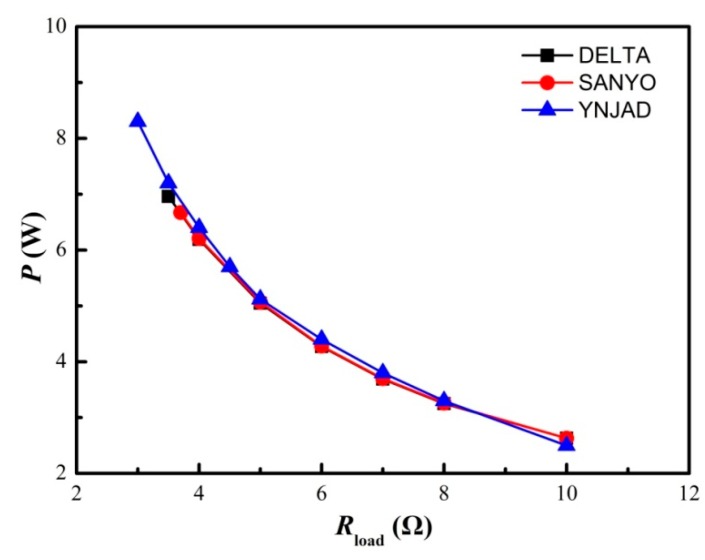
Influence of cooling fan selection on STEG output power performance (HS1 heat sink, Cod1 heat conducting plate and Cof1 configuration).

For the present STEG, electric power out is the most important consideration, i.e., it should be designed to extract as much electricity as possible. The power load feature for these three different cooling fans is shown in Figure 9. It is obvious that the maximum electric power output is closely related to the air flow rate. Choosing YNJAD produced higher maximum electric power (8.25 W) compared to that (about 7.0 W) of the other two types of cooling fan. Statistical analysis shows that the cooling air flow rate should be greater than 3.31 m^3^/h per TE module. Notice that the choice of heat-conducting plate thickness, heat sink dimensions and cooling fans are inter-dependent. Different types of cooling fans and heat sinks may be employed, and the present optimizations provide experimental data to select appropriate combinations of cooling fans and heat sinks.

### 3.7. Influence of the TE Module Configuration on the Startup Performance and Output Power

In line with the above discussions, the Cod1 heat-conducting plate, HS1 heat sink and YNJAD cooling fan were chosen to build the present STEG, yet the configuration of TE modules still remained to be optimized, i.e., different wiring configurations among the eight TE modules were to be optimized. Previous studies have found that minor differences in temperature level may cause a significant decrease in electric power output when placing the TE module in parallel upstream of the DC-DC converter [24]. On the other hand, wiring the TE modules in series results in large input voltage, which adversely affects the electric power output, which is caused by the transform efficiency of the DC-DC converter. The transform efficiency of the DC-DC converter is mainly affected by the ratio of the input voltage to the DC-DC converter and the output voltage (5.0 V). For the present STEG, the open-circuit voltage when wiring TE modules in series is much higher than that when wiring TE modules in parallel. This leads to a lower transform efficiency of the DC-DC converter, which results in less electric power output. Furthermore, the configuration of TE modules affects the startup performance, i.e., the input voltage has to be high enough to start the cooling fans during startup as soon as possible. Two configurations are shown in Table 7. Other configurations were tried and proved to be poor. The feasibility of Cof1 can be expected since two identical copper plates are installed opposite one another, suggesting each side has almost the same temperature. Therefore, four TE modules on each side can be wired in series, and the two groups then wired in parallel.

**Figure 10:**
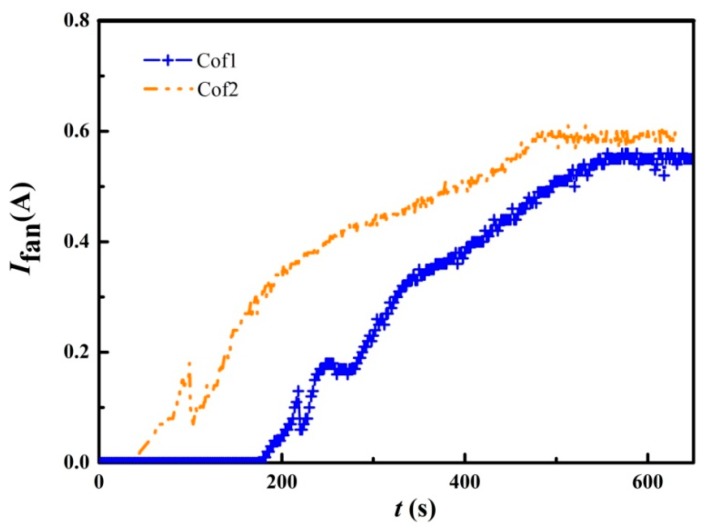
Influence of TE module configuration on STEG startup performance (HS1 heat sink, Cod1 heat conducting plate and YNJAD cooling fan).

**Figure 11:**
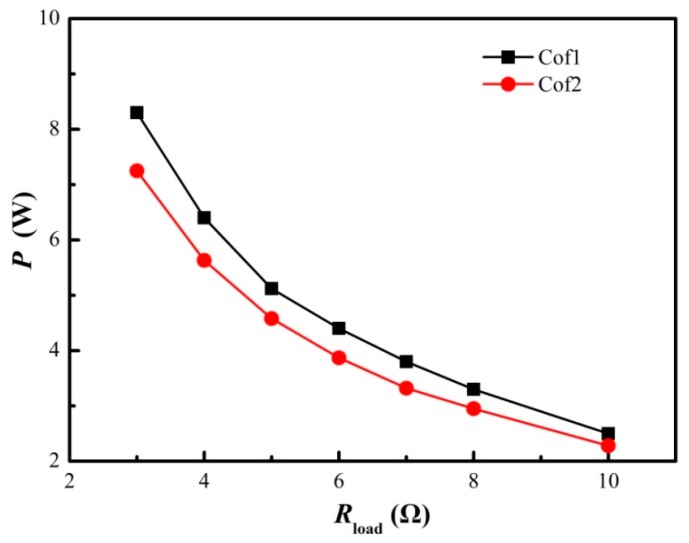
Influence of TE module configuration on STEG output power (HS1 heat sink, Cod1 heat conducting plate and YNJAD cooling fan).

The startup performances using these two different configurations are shown in Figure 10. The cooling fans started to work within 100 s for Cof2, while Cof1 needed about 216 s. This indicates that Cof2 should be considered when startup speed takes priority. Concerning the electric power output, shown in Figure 11, Cof1 has higher outputs than that of Cof2 under the same external resistance. i.e., the maximum electric power output of Cof1 is 1.05 W (14.5%) greater than that of Cof2 when *R*_load_ = 3 Ω, and the electric power output of Cof1 is 9.0% higher on average than that of Cof2 when 3 Ω ≤ *R*_load_ ≤ 10 Ω. On the other hand, the temperature difference of Cof1 was between 145 °C and 148 °C during the experimental tests, while it was between 151 °C and 153 °C for Cof2. The reason why the temperature difference is greater for Cof2 is related to the Peltier effect since less current was generated under this configuration. Therefore, it is obvious that Cof1 is better than Cof2 when electric power output is more important. For the pilot product using the present STEG, the Cod1 heat-conducting plate, HS1 heat sink, YNJAD cooling fan and Cof1 TE module configuration were finally chosen. Another advantage of the present STEG is the ease of augmenting the electric power output with little modification. Initial experiments showed that the present STEG can be modified to yield over 100 W of electricity power when water cooled while keeping the total weight under 20 kg.

## 4. Conclusions

In the present work, a stove-powered thermoelectric generator (STEG) was designed and optimized. The startup performance, power load feature and thermoelectric (TE) efficiency were studied in detail. Optimization was performed, including the effect of heat-conducting plate thickness, heat sink dimensions, cooling fan selection and TE module configuration. Several conclusions can be drawn based on the result analysis.

(1) For the present STEG, the measured maximum electric power is 14.79 W, and it decreases to 11.43 W when the output voltage is maintained at 5 V, of which 3.18 W is consumed by the cooling fans, while the remainder (8.25 W at 5 V) is ready to be used.
(2) For the present STEG, the TE efficiency is about 2.31% at a temperature difference of 148 °C, based on the heat flow method, which agrees well with the theoretically predicted value. This reveals that the low efficiency of the STEG is the major problem for large-scale applications.
(3) Optimizations indicate that the heat-conducting plate thickness, heat sink dimensions and cooling fan selection should be coordinated to increase the temperature difference and to control both the hot end temperature (below 250 °C) and the cold end temperature (below 70 °C). For the present type of STEG and TE module used, the heat flux, based on the cross-sectional area of the copper plate, should be around 2.9 × 10^5^ W/m^2^ per TE module, and the heat dissipation rate of the heat sink should be less than 745 W/m^2^, while the cooling air flow rate should be more than 3.3 m^3^/h per TE module.
(4) Experiments show that TE modules should be partly wired in parallel, which enhances the electric power output and ensures the self-startup of the cooling fans.
(5) The present STEG offers a combined heat and power design, and 625 W of clean and warm air can be used for heating purposes.

## Figures and Tables

**Table 1:** The errors of the measured parameters.

Parameter	Error (%)	Parameter	Error (%)
*U*	±0.5	*I*	±0.5
*P*	±0.5	*T*	±0.5
*ξ*_DC_	±1.0	*V*_ex,ave_	±2.0
*Q*_conv_	±3.0	*ξ*	±5.0

**Table 2:** The measured data of the flue gas.

Parameter	Value	Parameter	Value
*U*_out_ (V) *	4.98	*d* (mm)	120
*I* (A)	1.66	*T*_ex,ave_ (°C)	50.5
*I*_fan_ (A)	0.61	*T*_out,ave_ (°C)	58.0
*ξ*_DC_ (%)	77.3	*T*_∞_ (°C)	22.0
*V*_ex,ave_ (m/s)	1.40	*ξ* (%)	2.31

* The output voltage of the DC-DC converter for cooling fans is 5.2 V.

**Table 3:** Thermoelectric efficiencies of various STEGs.

Δ*T* (K)	TE Material	*ξ*	Cooling Method	References
200	Bi2Te3	2%	water cooled	[9]
150–200	Bi2Te3	4–5%	water cooled	[11,12]
150	Bi2Te3	3.2%	NDAC	[15]
148	Bi2Te3	2.31%	FDAC	present

**Table 4:** Thickness of the conducting heat plate for optimization.

No.	Thickness (mm)	Temperature Level
Cod1	1.5	<230 °C
Cod2	1.2	<190 °C
Cod3	1.8	<290 °C

**Table 5:** Characteristics of different heat sinks for optimization.

No.	L × W × H (mm)	Fin Area (m^2^)	Fin Number	Fin Gap (mm)	Fin Cross-Section Area (cm^2^)
HS1	215 × 88 × 56	0.42	19	2.98	61.3
HS2	215 × 98 × 31	0.34	29	2.20	68.6
HS3	215 × 70 × 32	0.35	25	1.64	59.2

**Table 6:** Different types of cooling fan for optimization.

Type No.	Flow Rate (m^3^/h)	Power (W)
SANYO	20.5	1.15
DELTA	20.1	0.94
YNJAD	26.5	1.59

**Table 7:** Different types of TE module configurations for optimization.

Cof. No.	Description
Cof1	4 modules in series, then the two groups in parallel
Cof2	8 modules in series

## Nomenclature

*c*_p_	heat capacity of air (kJ/kg-K)
*d*	diameter of the extensible aluminum tube (mm)
*I*	load current (A)
*I*_fan_	cooling fan current (A)
*L*	length of thermo-element (mm)
*m*_ex_	exhaust air flow rate (kg/s)
*n*	ratio of electrical resistivity (mm)
*N*	number of TE module
*P*	power (W)
*P*_fan_	power consumed by cooling fans (W)
*P*_max_	maximum electricity power (W)
*P*_tot_	total electricity power (W)
*P*_TE_	total electricity generated per TE module (W)
*Q*_in_	total amount of heat conduction through copper plates (W)
*Q*_out_	amount of heat dissipation by heat sinks (W)
*r*	thermal contact
*R*_load_	load resistance (Ω)
*T*	temperature (°C)
*T*_c_	hot end temperature (°C), *T*_c_ = *T*_2_
*T*_ex,ave_	averaged exhaust air temperature (°C), averaged *T*_4_
*T*_h_	hot end temperature (°C), *T*_h_ = *T*_1_
*T*_out,ave_	averaged outlet air temperature (°C), averaged *T*_3_
*T*_∞_	atmosphere temperature (°C)
Δ*T*	temperature difference (°C), Δ*T* = *T*_h_ − *T*_c_
*U*	voltage (V)
*U*_in_	Input voltage to the DC-DC converter (V)
*U*_out_	output voltage of the DC-DC converter (V)
*V*_ex,ave_	averaged exhaust air velocity (m/s)
*w*	ratio of ceramic thickness to that of thermo-element
*Z*	thermoelectric figure-of-merit (1/K)
*ρ*_ex,ave_	averaged exhaust air density (kg/m^3^)
*ξ*	thermoelectric efficiency (%)
*ξ*_DC_	transform efficiency of the DC-DC converter (%)
**Subscripts**	
1–4	The number of the thermocouple
**Abbreviation**	
CHP	combined heat and power
Cod	heat conduction plate
Cof	configuration
DAQ	data acquisition
DELTA	a type of cooling fan, brand “DELTA”
FDAC	forced draft air cooled
HS	heat sink
NDAC	natural draft air cooled
SANYO	a type of cooling fan, brand “SANYO”
TE	thermoelectric
YNJAD	a type of cooling fan, brand “YNJAD”
